# Alpha desynchronization/synchronization during working memory testing is compromised in acute mild traumatic brain injury (mTBI)

**DOI:** 10.1371/journal.pone.0188101

**Published:** 2018-02-14

**Authors:** Xianghong Arakaki, Michael Shoga, Lianyang Li, George Zouridakis, Thao Tran, Alfred N. Fonteh, Jessica Dawlaty, Robert Goldweber, Janice M. Pogoda, Michael G. Harrington

**Affiliations:** 1 Neurosciences, Huntington Medical Research Institutes, Pasadena, California, United States of America; 2 Biomedical Imaging Lab, University of Houston, Houston, Texas, United States of America; 3 Emergency Department, Huntington Hospital, Pasadena, California, United States of America; 4 Columbus Biometrics, LLC, Reno, Nevada, United States of America; University of Electronic Science and Technology of China, CHINA

## Abstract

Diagnosing and monitoring recovery of patients with mild traumatic brain injury (mTBI) is challenging because of the lack of objective, quantitative measures. Diagnosis is based on description of injuries often not witnessed, subtle neurocognitive symptoms, and neuropsychological testing. Since working memory (WM) is at the center of cognitive functions impaired in mTBI, this study was designed to define objective quantitative electroencephalographic (qEEG) measures of WM processing that may correlate with cognitive changes associated with acute mTBI. First-time mTBI patients and mild peripheral (limb) trauma controls without head injury were recruited from the emergency department. WM was assessed by a continuous performance task (N-back). EEG recordings were obtained during N-back testing on three occasions: within five days, two weeks, and one month after injury. Compared with controls, mTBI patients showed abnormal induced and evoked alpha activity including event-related desynchronization (ERD) and synchronization (ERS). For induced alpha power, TBI patients had excessive frontal ERD on their first and third visit. For evoked alpha, mTBI patients had lower parietal ERD/ERS at the second and third visits. These exploratory qEEG findings offer new and non-invasive candidate measures to characterize the evolution of injury over the first month, with potential to provide much-needed objective measures of brain dysfunction to diagnose and monitor the consequences of mTBI.

## Introduction

The incidence of mild traumatic brain injury (mTBI), or concussion, is estimated to be over 6 per 1,000 people each year [1]. Patients with mTBI are predisposed to other injuries, particularly before all symptoms resolve [2]. Repeated mTBI increases the risk for subsequent neurological diseases, such as dementia, depression, and migraine [3]. The economic burden of mTBI rivals that of moderate and severe brain injuries due to loss of work productivity and forced early retirement [4]. It is difficult for physicians to rigorously diagnose mTBI, mainly from lack of objective markers to identify and quantify the injury.

Frontal lobe executive dysfunction is almost universally present in acute mTBI and usually persists for several hours or longer [5], but impaired executive function is hard to recognize. In attempts to detect dysfunction, abnormal electroencephalogram (EEG) or magnetoencephalogram (MEG) features have been described in the frontal lobe [6–8] shortly after injury. Challenges that involve or impact executive function activate a combination of top-down and bottom-up information processing pathways. When an external stimulus (eg. an image or sound) elicits perceptual representation (sensation), bottom-up processing occurs. When the cognitive process is influenced by higher mental functions such as motivation or expectation, top-down processing occurs [9, 10]. Top-down activities are mediated by alpha oscillations, and can be assessed by task-related executive functions [11, 12].

Working memory (WM) as a core executive function refers to the cognitive ability to transiently store and manipulate information in real time [9]. WM can be easily assessed by (visual) N-back testing, whereby, for example, letters are displayed on a computer screen and the patient is asked to press a button when a target letter appears (0-back), or if the letter that appears on the screen is the same one presented two screens back (2-back). Brain imaging can reveal the brain networks that are activated during N-back WM tests [10, 11]. Functional magnetic resonance imaging (fMRI) studies have shown that the ability to increase activation in WM circuitry is impaired in mTBI patients [12]. However, the mechanisms by which brain resources are allocated and integrated to support WM functions, and the extent these processes are compromised in mTBI, are still unclear.

Brain activity, demonstrated by intra- or inter-regional interactions, is thought to result from neuronal synchronization and neural oscillations. EEG recordings during WM testing can identify cerebral oscillatory dynamic changes in the WM network, and so are well-suited to the study of mTBI. Oscillatory activity in the alpha band (8–12 Hz) is the dominant oscillation in human brains and is the only activity that responds to a stimulus with both decrease and increase in power, such that the alpha frequency event-related desynchronization (ERD) is followed by event-related synchronization (ERS) [13]. Alpha ERD is related to memory storage and ERS to memory retention, [14, 15], and so are the focus of our study. Alpha frequency oscillations represent thalamocortical interactions and are essential for information selection and storage functions, including attention and WM tasks [13, 16]. They relate to encoding and manipulation of spatial representations in WM [17] and play an important role in top-down control mechanisms [18]. Pathology can disrupt normal alpha synchronization physiologies in many ways. Alpha ERD during the WM task was reported to be lower in people with a high intelligence quotient, supporting a higher “neural efficiency” [19–21]. Alpha ERD during WM is associated with fronto-parietal network activity, supporting the alpha oscillation relationship to top-down network interactions [22], as shown in concurrent EEG and fMRI recordings [16]. Similar associations have been found in attention deficit/hyperactivity disorder (ADHD) studies [22, 23].

Comparison between evoked and induced activity has been overlooked in previous EEG WM studies [24]. Evoked or phase-locked activity is both time- and phase-locked to the stimulus and is directly driven by the eliciting event. Induced or non-phase-locked activity is time-locked, but not phase-locked to the stimulus and reflects the dynamics that control interactions within or between brain structures [24], representing frontal lobe function or top-down mechanisms [25–28].

Our study aimed to explore how cerebral oscillatory activities change in an acute/subacute mTBI setting. We analyzed evoked and induced EEG activity in a visual N-back WM paradigm to examine differences in activity changes between mTBI patients and trauma controls. We specifically focused on induced and evoked activity in the 8–12 Hz range in acute mTBI. In addition to overall alpha power comparisons between mTBI and control groups, we also explored alpha power on specific sensors based on symptoms and neurometabolic changes at different stages after mTBI as reported in the literature [2, 29–33]. We show that induced and evoked alpha ERD or ERS are abnormal at different sensors or brain regions at different times during the month after mTBI.

## Materials and methods

We designed the study to investigate the neural correlates of mTBI symptom evolution that we would expect during the first month after injury [2, 29–34]. The first time point, within 5 days of injury, was selected to measure WM performance during the acute phase of cognitive deficit, when changes in symptom scales, balance and neurocognitive testing [29], and neurometabolism [32, 33] would be expected. The second time point, 2 weeks after injury, was chosen to measure WM performance when most cognitive functioning begins to normalize [29, 32]. The third time point, one month after injury, was chosen to measure the expected continuing resolution of the mTBI-induced neurocognitive symptoms and to assess any possible residual learning impairment (compared to previous assessments and to peripheral trauma controls) [2, 33, 34].

### Patients

The experimental protocol and informed consent documents were approved by an Institutional Review Board (Quorum Review IRB). All patients signed informed consents before participating in the study. Trauma patients between 18–50 years of age with either mTBI (diagnosed by emergency department physicians) or non-head mild traumatic injury (controls) were recruited from the emergency department of Huntington Hospital in Pasadena, CA. All mTBI patients had no evidence of skull fracture, brain laceration, or intracranial hemorrhage by computed tomography (CT) scan. Controls had minor non-head trauma not requiring surgery beyond skin sutures and dressings and had the ability to comply with the study protocol. Exclusion criteria included previous TBI, any significant major end-organ pathology such as heart disease or cancer; pregnancy, illicit drug use, sedative medications, alcohol abuse, and injuries or conditions that could affect study compliance.

Thirteen mTBI and seven trauma controls were recruited in this pilot study. Patients from the two groups were similar in age, gender distribution, years of education, body mass index (BMI), and handedness (Table 1). Injury type and locations are shown in Table 2. For the mTBI group, causes of injury were vehicle accidents (n = 6), fall-related accidents (n = 4), sports injuries (n = 2), and bumping (n = 1). For the control group, causes of injury were vehicle accidents (n = 1), fall-related accidents (n = 4), sports injuries (n = 1), and dog bite (n = 1). There were missed visits for some patients due to conflict of scheduling, as shown in detail in Table 3.

**Table 1:** Baseline characteristics of patients.

		mTBI (n = 13)	Controls (n = 7)	p-value
Mean Age (SD)	Mean (SD)	26.4 (7.0)	27.6 (6.0)	0.68*
Gender [n (%)]	Female	7 (54%)	4 (57.1%)	0.89^#^
	Male	6 (46%)	3 (42.9%)	
Mean Education (SD) (yrs)		14.2 (2.8)	13.9 (1.2)	0.74*
Mean BMI (SD) (kg/m^2^)		29.0 (5.8)	28.40 (4.1)	0.78*
Handedness [n (%)]	R	11 (85%)	6 (86%)	1.00^#^
	L	2 (15%)	1 (14T%)	
SAC score		25 (3.2)	NO SAC scores	

Abbreviations: BMI, body mass index; R/L, right/left; SAC, Standardized Assessment of Concussion; SD, standard deviation.

* Two-tailed t-test

^#^ Fisher's exact test.

**Table 2:** Injury type and location.

Mild traumatic head injury (mTBI)					Non-head trauma controls				
Pt. ID	M/F	age	injury location	injury type	Pt. ID	M/F	age	injury location	injury type
mTBI007	M	18	head left front (F7/F3/T3/C3)	motor cycle	mTBI015	F	37	right forearm, cast on	fell on floor
mTBI014	M	23	head right front (F4-F8), whiplash	car accident	mTBI016	M	22	left shoulder	skate board
mTBI011	M	36	head, whole right side	fall off stairs, head on concrete	mTBI017	M	25	right ankle sprain 7/10	during playing basketball
mTBI013	F	28	right leg, head	fall off stairs	mTBI019	M	24	left knee and thigh	motor cycle accident
mTBI018	M	21	right side body and head	motorcycle crash	mTBI036^#^	M	30	left arm dog bite	dog bite while protecting his own dog
mTBI020	F	37	head back/left	softball hit	mTBI038	F	25	left foot	run over by car
mTBI031	F	35	back of head	car accident	mTBI040	F	22	both legs	fell and hit on legs
mTBI034	M	21	front left side	skateboard fall	mTBI041	F	36	feet and ankles	dropped log on feet and ankle twisted
mTBI035	F	25	Whiplash	car accident					
mTBI037	M		back right side of head	hit by 2x4 wood					
mTBI039	M	18	whiplash (front and both temporal headache)	car accident					
mTBI042	F	28	tree feel on left side of head	tree fell on head					
mTBI043	F		front left side of head	hit cabinet					

^#^: patient's head was too big for EEG headset.

**Table 3:** Mean (SD) response accuracy (ACC) and response time (RT) in N-back WM by visit.

	mTBI			Controls		
	Visit 1	Visit 2	Visit 3	Visit 1	Visit 2	Visit 3
0-back						
N	11	11	13	7	5	5
ACC	0.97 (0.03)	0.97 (0.04)	0.96 (0.05)	0.97 (0.05)	0.97 (0.02)	0.98 (0.02)
RT (ms)	466.4 (48.7)	446.8 (56.8)	444.9 (65.5)	492.3 (100.9)	457.6 (55.5)	488.0 (74.8)
2-back						
N	11	12	13	7	5	5
ACC	0.84 (0.07)*	0.89 (0.07)	0.88 (0.12)	0.91 (0.05)	0.89 (0.12)	0.92 (0.08)
RT (ms)	553.3 (130.6)	513.2 (95.3)	524.7 (154.9)	640.4 (206.4)	523.3 (145.0)	574.0 (189.6)

* p = 0.03. There are missing visits for some patients.

### Procedures

The brain cognitive challenge, or N-back WM test (N = 0, 2 to reflect the workload level of the task), was administered using E-prime software (Psychology Software Tools, Inc., Sharpsburg PA) on a Dell Precision T5610 with a 20 inch screen. Although different types of stimuli can be used for WM, in this study we used letters [35].

**Fig 1:**
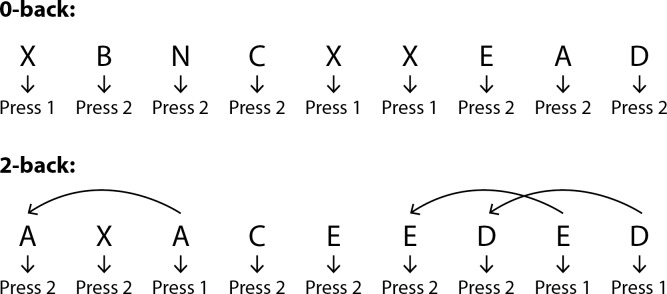
Experimental instructions given to all patients. Letters will flash on the screen one at a time. For 0-back, when you see X, press 1 with index finger; otherwise, please press 2 with middle finger; For 2-back, if the letter that appears on the screen is the same as the letter you saw two letters ago, press 1 with index finger; otherwise, please press 2 with middle finger.

Overall, patients were comfortably seated in front of a computer screen at a distance of approximately two feet, and were instructed and tested for 0-back, then for 2-back. Instructions were given to each patient before each workload. Uppercase letters were displayed on the screen one at a time for 0.5 seconds, separated by a 2.4-second interval. All patients were asked to use the right hand to respond, regardless of handedness. For 0-back, patients were asked to look for the target letter “X”, and press 1 using their index finger when “X” appeared on the screen, or press 2 for all other letters, using their middle finger. For 2-back, patients were required to remember letters they saw previously. If the letter that appeared on the screen was the same as the letter shown two letters ago, patients were required to press 1; otherwise, they were to press 2 using the same fingers as before. Fig 1 illustrates the instructions given to each patient for this WM task. Patients were presented with instructions displayed at the start of each workload. First, all patients were required to complete a 1.5-minute practice block that included 30 trials at the beginning of each workload. At the end of practice block, response accuracy feedback was provided, and each patient could choose to redo the practice block, or continue to do task blocks. Second, after the practice block indicated that task instructions were clear and understandable for each patient, they were asked to proceed to do task trials, which included 3 blocks of 30 trials for each block and for each workload. The n-back task took about 12–25 minutes to complete, depending on each patient’s performance.

### EEG recordings

Continuous EEG activity was recorded while patients engaged in the memory challenge tasks, using a 21-sensor, dry electrode system (Quasar Wearable Sensing, DSI-24, San Diego, CA). Sensor arrangement followed the international 10–20 system and were placed approximately at the locations Fp1, Fp2, F7, F3, Fz, F4, F8, T3, C3, Cz, C4, T4, T5, P3, Pz, P4, T6, O1, O2, M1, and M2. All activity was referenced to Pz. Sensor impedances were kept below 1 MOhm. EEG signals were sampled at 300 Hz, and bandpass filtered between 0.003–150Hz. Electrooculographic (EOG) and electrocardiographic (ECG) activity was recorded using two pairs of auxiliary sensors. The time of presentation of the letter stimuli, the patients’ responses, and the type of test (0- or 2-back) were encoded with electronic pulses, which were saved with the EEG data for off-line analysis.

### Data processing

The behavioral responses were summarized by accuracy (ACC) and response time (RT). ACC was calculated as the percentage of correct responses out of the total number of trials. RT was defined as the latency between stimulus onset and patient response.

All datasets underwent the same processing regardless of clinical classification of the patient, using EEGLAB version 13.4.3b [36] running in MATLAB R2014a (The MathWorks, USA) and custom software developed in-house. All EEG signals were re-referenced to the mean of two mastoid sensors (M1 and M2). The continuous EEG recordings were segmented into epochs, using the stimulus onset as a reference, including 500 ms before and 2500 ms after the stimulus onset. Individual epochs were baseline-corrected and bandpass filtered between 2 and 30 Hz. Furthermore, independent component analysis (ICA) [36] was used to remove eye blinks and cardiac and other muscle artifacts. Also, epochs that contained large artifacts, i.e., activity greater than three standard deviations (SDs) from the mean of a specific sensor, were rejected.

The epoched EEG data were decomposed into a time-frequency (TF) representation with logarithmic scaling between 2 and 30 Hz from fast Fourier transform and via Morlet wavelet [$e^{i2\pi tf}e^{-t^{2}/{2\sigma }^{2}}$] convolution with the single-trial EEG data performed in the frequency domain, followed by the inverse fast Fourier transform[27, 37]. In order to remove scale differences between individuals, all power values in the TF representation were normalized by decibels to the baseline power computed as the average power from -400 to -100 ms prestimulus at each frequency band [$dB\;power=10*log10(\frac{power}{baseline})$]. Based on the TF plots and published data, alpha ERD (range 200–800 ms, 8–12 Hz) and alpha ERS (range 1000–2500 ms, 8–12 Hz) were then extracted for comparison, including total power, non-phase-locked power (induced power), and phase-locked power (referred to as phase-locked to stimulus onset, or evoked power), which were acquired by the following steps. First, ERP was calculated by averaging all trials. Second, induced power was calculated as described as above from the differences between each trial and ERP calculated on time domain. Third, evoked power was calculated by subtracting the non-phase-locked (induced) from the total power [27, 37]. This was done separately for each sensor, condition, and patient.

### Hypothesis generation

We based the following hypotheses on literature describing mTBI symptoms and neurometabolic changes [2, 30, 31, 34, 38–42].

#### Hypothesis 1

The first 5 days after mTBI is the acute phase of cognitive deficit associated with increased metabolic demands on the brain [30, 31]. Because cognitive function involves the frontal lobe, and reported acute symptoms indicate a “top-down” executive function impairment, we hypothesized that mTBI patients at the initial visit will have altered induced alpha ERD at the Fz sensor (located at the midline of the frontal lobe) during the 0-back task, i.e., even when the work load is minimal. The frontal midline sensor Fz was chosen for “top-down” function assessment based on previous auditory ERP and EEG alpha oscillation on visual facial preference studies [38].

#### Hypothesis 2

Previous cognitive evaluations and EEG studies indicate learning impairment in mTBI patients [2, 34]. We hypothesized that our longitudinal WM data would show group differences in learning, especially when using the more challenging 2-back task, and that these differences would be greatest 30 days post-injury (i.e., at the third visit). Learning is part of top-down executive function, measured by induced or non-phase-locked activity [39–41]. WM is mediated by the orbitofrontal cortex, an area that can be assessed by Fp1 and Fp2 sensors [42]. If controls, but not mTBI patients, were able to learn over the 30-day study time period, we would expect alpha ERD at the Fp1 and Fp2 sensors to decrease over visits in controls but remain stable in mTBI patients, or alpha ERD at Fp1/Fp2 sensors at the third visit during 2-back test differs between mTBI and control patients.

### Statistical methods

Evoked and induced alpha power measurements were analyzed by averaging individual sensors within and across patients and visits to derive summary statistics for the following variable clusters: frontal (Fz, F3, F4), central (Cz, C3, C4), parietal (Pz, P3, P4), left lateral (F7, T3, T5), right lateral (F8, T4, T6), and occipital (O1, O2). Group comparisons on patient baseline characteristics were done using two-sided t-tests or Fisher’s exact tests. Longitudinal analyses were done using general linear mixed models with group (mTBI or Control) and visit (1, 2, or 3) as fixed effects and patient as a random effect. A term for the interaction between group and visit was included to evaluate varying group effects over visit. Group comparisons within visits were done using two-sided t-test. As this was an exploratory, hypothesis-generating study, no adjustments were made for multiplicity. A significant level of 0.05 was used for all tests. Analyses were done using PRISM v6.07 (GraphPad) and SAS v9.4 (SAS Institute, Cary, NC).

## Results

### Behavioral performance (ACC and RT)

As seen in Table 3, for the 0-back test, neither ACC nor RT was significantly different between the mTBI and control patients. Considering all of the data simultaneously (but ACC and RT separately), there are no statistical differences between mTBI and control patients in ACC for the 2-back test.

### Induced alpha ERD at Fz sensor, 0-back test, first visit

**Fig 2:**
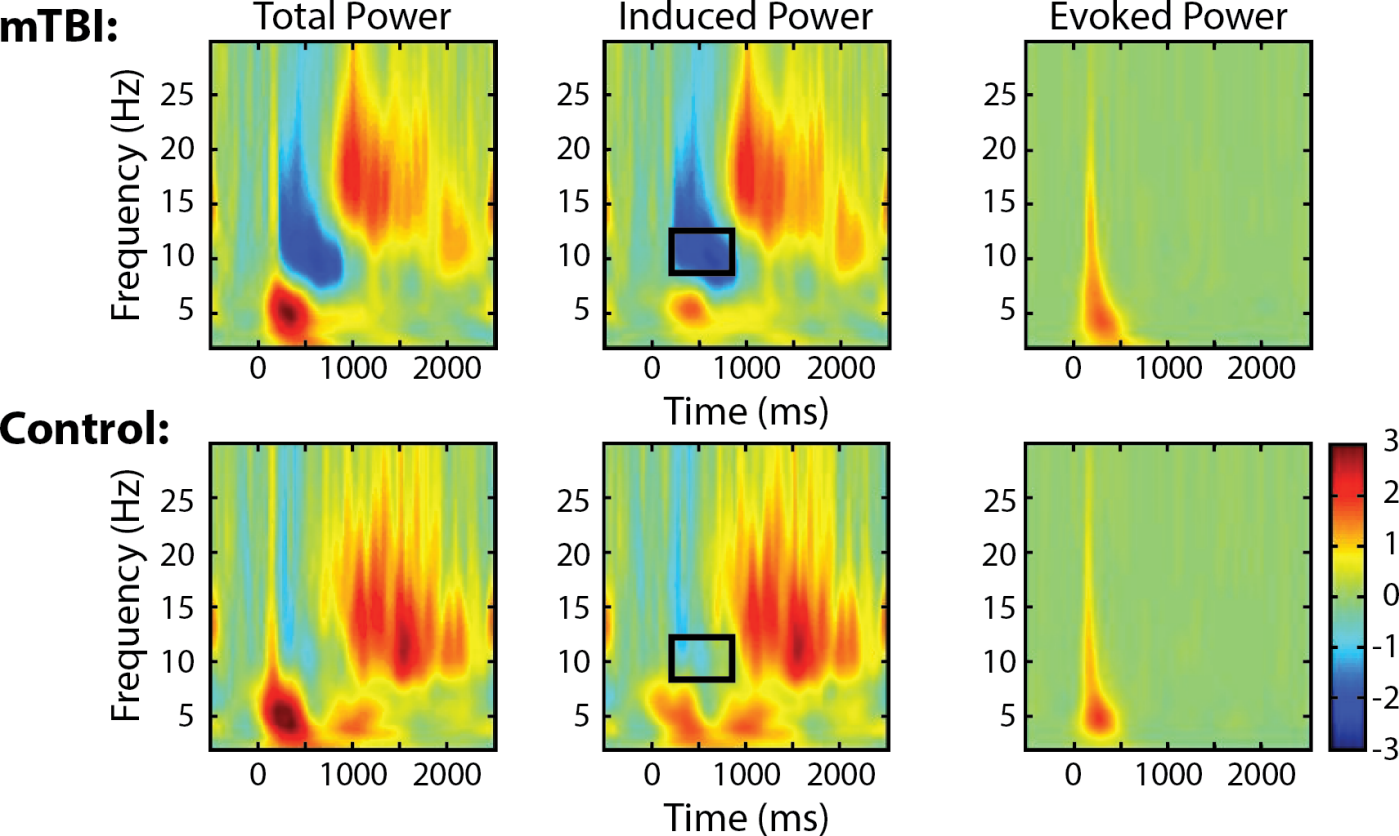
Time-frequency plots (Fz sensor) of mean 0-back test, first visit. Column 1 shows total power, column 2 induced power, and column 3 evoked power. The rectangles on the induced power plots locate areas of excessive alpha ERD in mTBI (N = 13) vs. control (N = 7) patients.

Fig 2 shows a comparison (mTBI vs. controls) of time frequency plots of mean induced alpha power of EEG at the Fz sensor during the 0-back test at the first visit. Despite “normal” behavioral performance measures (Table 3), total power of alpha ERD appeared to be greater (more negative) in the mTBI group compared to controls, as evidenced in Fig 2, Column 1. This difference is seen to derive from the induced rather than the evoked power (Fig 2 and Table 4; p = 0.08 for interaction between group and power type, p = 0.08 and 0.06 for total power and induced power, respectively). The induced alpha ERD differences between mTBI and controls appeared to differ only marginally (Table 4, p = 0.06).

**Table 4:** Comparison of induced, evoked, and total alpha ERD between mTBI patients and controls during 0-back test at first visit.

	mTBI (n = 11)		Control (n = 7)		P value
	Mean	SD	Mean	SD	
Induced power	-1.78	2.14	0.22	1.17	0.06
Evoked power	0.21	0.19	0.16	0.16	0.57
Total Power	-1.57	2.16	-0.05	1.28	0.08

When comparing induced alpha ERD at the Fz sensor during different workloads at the first visit, i.e., 0-back vs. 2-back, control patients’ induced alpha ERD was numerically (but not significantly) lower during 2-back (-1.78+/-2.38) compared to 0-back (-0.22+/-1.17), while mTBI patients’ induced alpha ERD during 0-back and 2-back was numerically similar (-2.01+/-2.50 during 2-back vs. -1.78+/-2.14 during 0-back).

### Induced alpha ERD at Fp1/Fp2 sensors, 2-back test

**Fig 3:**
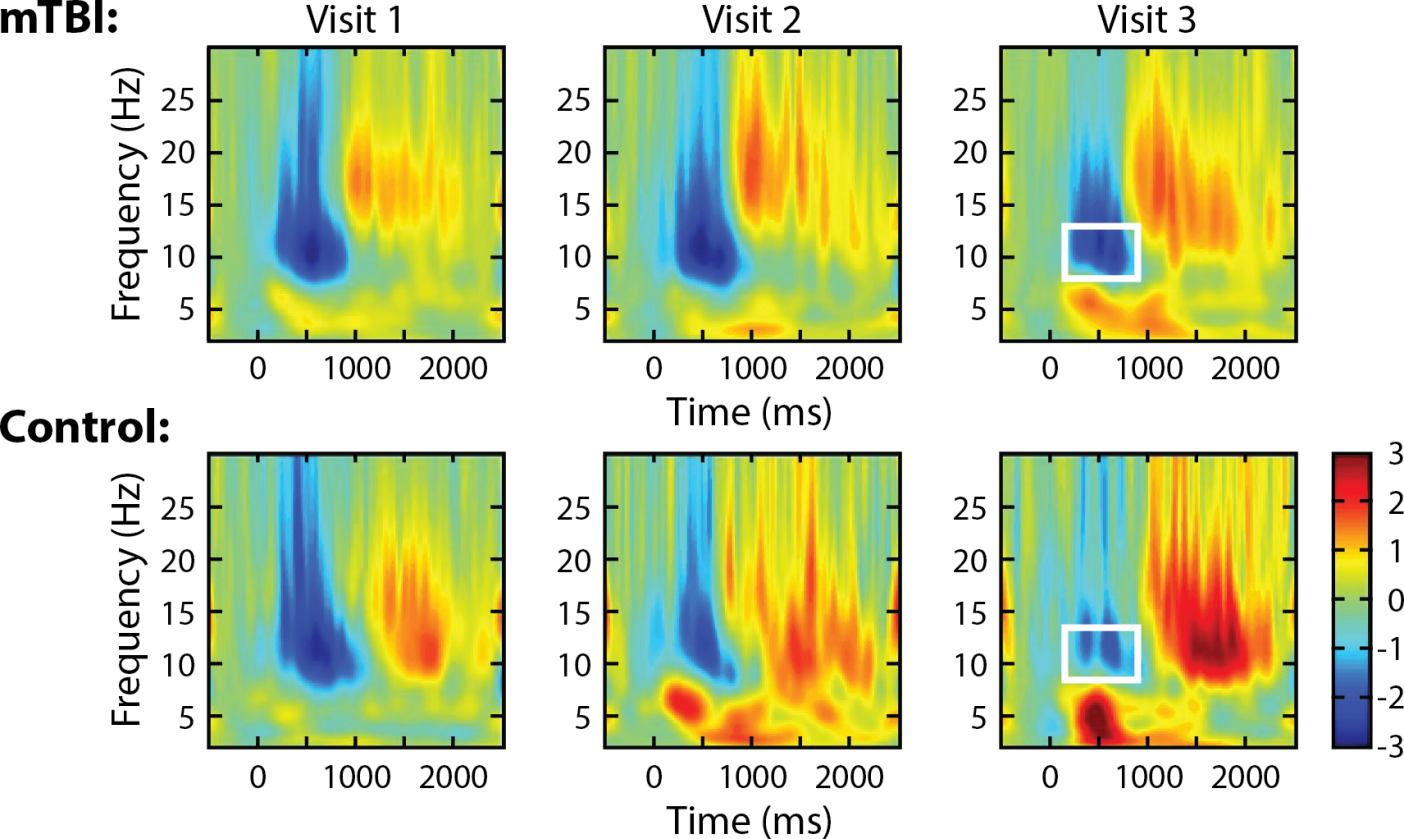
Time-frequency plots (Fp1 sensor) of mean 2-back test, induced power, by visit and group. Excessive alpha ERD (white rectangles) remained in the mTBI group compared to controls.

Fig 3 compares mTBI and control patients on time frequency plots of mean induced alpha power of EEG at the Fp1 sensor during the 2-back test for each of the three study visits (data for Fp2 sensor was similar, not shown). The figure suggests that controls used less alpha ERD as time passed after trauma while alpha ERD in the mTBI patients remained elevated (more negative) over time, though the effect of time did not statistically differ by group, nor was “visit” a significant main effect within the control group.

**Fig 4:**
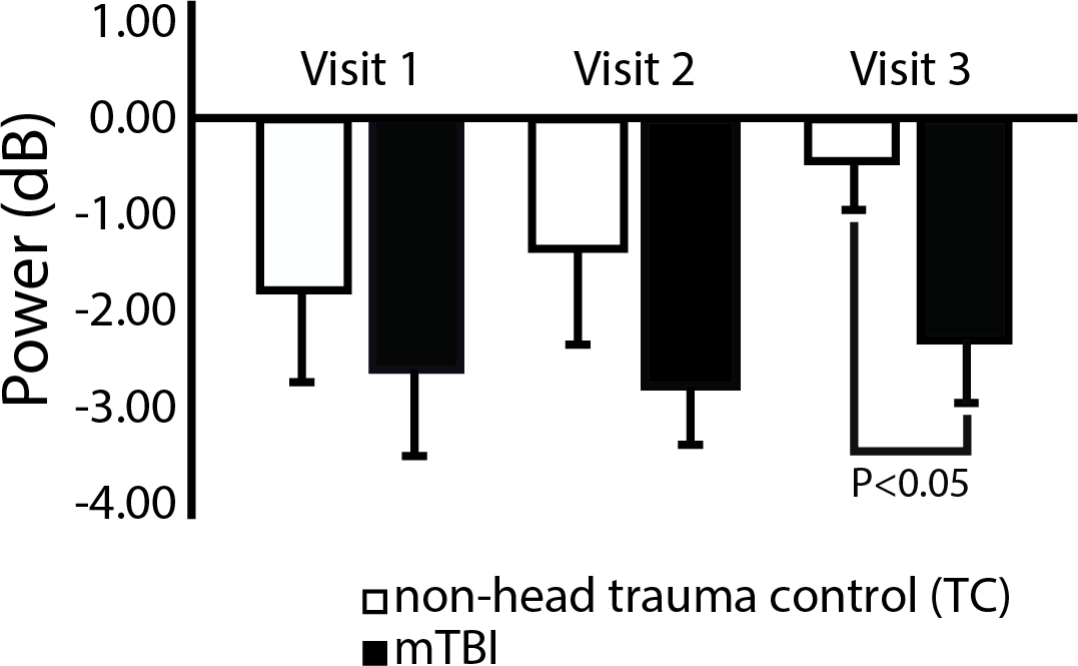
Mean (SE) induced alpha ERD from Fp1 sensor, 2-back test, by visit and group. Induced alpha ERD at the third visit was significantly different between mTBI and control group. The alpha ERD in the control group appeared to lessen with successive visits, while alpha ERD in the mTBI group appeared to remain elevated (more negative) over all visits.

Analysis by visit revealed a significant difference between groups at the third visit, with alpha ERD power less negative in controls compared to mTBI patients (p = 0.04, Fig 4. Data for Fp2 sensor was similar, not shown).

### Induced alpha power at all sensors, all visits

Summary of induced alpha ERD and ERS power for all sensors at all visits during 0-back or 2-back are shown in S1–S4 Tables.

### Evoked alpha power at all regions, all visits

For the 0-back test at the parietal location, for ERD there was an interaction between group and visit (p = 0.02). Analysis stratified by visit revealed a group difference at the second visit only (p = 0.03), with controls measuring higher (less desynchronization) than mTBI patients (Table 5), largely due to increased ERD (less desynchronization) in controls compared to mTBI patients (p = 0.04 for “visit” effect).

**Table 5:** Evoked parietal alpha ERD during 0-back by visit.

Parameter	---------------mTBI---------------			-------------Controls-------------			Group
	n	Mean	SD	n	Mean	SD	p-value
ERD PARIETAL Visit 1	11	0.14	0.11	7	0.08	0.07	0.20
ERD PARIETAL Visit 2	11	0.09	0.10	5	0.22	0.11	**0.03**
ERD PARIETAL Visit 3	13	0.12	0.12	5	0.10	0.14	0.76

For the 2-back test at the parietal location for ERS, controls were more synchronized than mTBI patients at the third (p = 0.04) visits (Table 6).

**Table 6:** Evoked parietal alpha ERS during 2-back by time.

Parameter	---------------mTBI---------------			-------------Controls-------------			Group
	n	Mean	SD	n	Mean	SD	p-value
ERS PARIETAL Visit 1	11	0.01	0.05	7	0.01	0.05	0.84
ERS PARIETAL Visit 2	12	0.00	0.05	5	0.04	0.05	**0.13**
ERS PARIETAL Visit 3	13	0.00	0.04	5	0.04	0.04	**0.04**

There were no significant group differences among all other regions and visits, or for induced alpha power.

## Discussion

Our study observed identifiable qEEG changes between mTBI patients and non-head-trauma controls in a dynamic setting at times coincident with reports of evolving symptoms in the acute and subacute period after injury [2, 29–33]. Our study supports the hypothesis that induced frontal alpha power was excessive within 1 month after mTBI. The most significant difference we observed was that evoked parietal alpha power 2 weeks after injury in mTBI patients was more negative compared to trauma controls. Our findings suggest the potential for non-invasive measures for acute mTBI patients in the clinic. A strength of the study was the nature of the control group, often omitted in mTBI studies.The choice of the control population was purposeful: We used controls who experienced the stress of trauma coupled with an ER visit to minimize the possibility that EEG changes resulted from pain or other symptoms associated with peripheral trauma rather than specifically from head injury. Causes of injury among our mTBI patients were consistent with what has been reported most frequently among adults, namely vehicle accidents and falls [43].

Consistent with clinical acute/subacute symptom evolution [2, 29–33], we observed an improvement of WM behavioral performance after mTBI. N-back behavioral performance (ACC and RT) was similar in mTBI patients and controls one month post-injury, in agreement with other reports [44–46]. There were no significant differences in RT between controls and mTBI patients at any visit, although mean RT tended to be shorter in mTBI patients compared to controls, also consistent with the literature [45, 46]. This might be because of the relatively young age of the cohort we studied, similar to the age range of previous reports [45, 46]. In young age, relatively higher cognitive reserve and WM capacity can compensate impairment from mTBI, therefore behavioral performance remains similar; however, the cognitive reserve and WM capacity declines in older age [47, 48], possibly from reduced distraction control during WM in older adults [49]. Therefore, we can speculate that the behavioral performance in an older population can be significantly different after mTBI because of less cognitive reserve. Although at the present stage of our research we cannot correlate brain regions with WM performance, a previous fMRI study indicates that right prefrontal cortex appears to be critical for WM network functioning and performance [45].

Two existing hypotheses are supported by our qEEG results and one new hypothesis has been generated. Our data indicate that alpha power, specifically induced and evoked alpha power from N-back WM testing, is different between mTBI and control patients, suggesting that alpha ERD/ERS is potentially useful in the diagnosis of mTBI.

Hypothesis 1: Induced alpha ERD at Fz sensor is marginally different (p = 0.06) between mTBI and control groups. For 0-back testing at the first visit, we found that induced alpha ERD during encoding tended to be greater (more negative) in mTBI patients compared to controls, indicating lower neural efficiency and impaired WM capacitiy after mTBI [21, 50]. Because the behavioral responses of mTBI patients during the 0-back task were similar to those of controls, this excessive frontal alpha ERD during a simple task may imply a compensatory attentional response and is a likely indicator of weak top-down control and lack of attention during WM encoding after mTBI. This observation is consistent with published complaints of mTBI patients regarding their inability to focus or “inattention” which, to date, lacks an objective clinical measure [29]. The Fz alpha ERD during 2-back was similar between mTBI patients and controls. The different workload results support that qEEG-based workload assessment can be used to indicate the resilience of the WM network [51]. Further investigation to examine if the workload effect is revealed in other sensors besides Fz may be informative, and will be addressed in future studies.

Hypothesis 2: Induced alpha ERD at Fp1/Fp2 sensors at the third visit during 2-back tasks differs significantly between mTBI and control groups. Although the N-back is used for testing WM rather than learning, our longitudinal data afforded us the opportunity to examine N-back processing changes over time to evaluate learning effects. Alpha ERD during 2-back tended to decrease in controls from the first to the third visit (though not significantly), but seemed to remain unchanged in mTBI patients over this time period; these patterns are consistent with a learning effect in controls, but suggest a learning impairment in mTBI patients. The greater induced alpha ERD of mTBI patients compared to controls during 2-back at the third visit also indicates lower WM capacity after mTBI, consistent with previous qEEG evidence of greater ERD in people with lower WM capacities [21, 50]. Lower WM capacity could contribute to learning impairment after mTBI, and both might contribute to long-term cognitive deficits (specifically regarding impaired attention and memory) after mTBI [52]. This mTBI-induced learning deficit reflects reduced brain reserve. An important consequence of mTBI impaired learning might be reduced risk aversion, which may contribute to mTBI patients being three times more likely to sustain another mTBI compared to controls [53].

The new hypothesis generated by our analysis is based on our finding that alpha ERD/ERS differs between mTBI and control groups two weeks after injury. In an analysis of evoked power, we observed that alpha ERD in the parietal area of controls was significantly higher than in mTBI patients but only during the 0-back test at the second visit. This difference is largely due to a significant increase of evoked alpha ERD in controls at visit 2. Whether this is a spurious finding or a real group effect during their second visit is worth further study. Analysis of evoked alpha power during 2-back demonstrated that parietal alpha ERS was significantly higher in controls compared to mTBI patients at the third visits, which indicates that a deeper evoked alpha power defect persisted with higher workload for a more prolonged period in these patients.

The most common cognitive symptom after mTBI is feeling “slowed down”, “in a fog”, or “dazed,” [54, 55] indicating abnormal sensory perception assessed by evoked activity [56]. However, there are no published reports about how the “foggy” symptoms evolve after injury, especially in the acute phase.

Evoked alpha power contributes to visual perception [56]. These abnormal parietal evoked alpha ERD/ERS measures after mTBI may correlate with the “dazed” feelings reported after acute mTBI, a symptom that usually resolves within a month after injury [54–56]. The alpha ERD is closely related to memory storage [14], and ERS is associated with retention [15]. Therefore, our results support that mTBI might impair information storage for a low-load task and impair information retention for a higher-load task 2 weeks post-injury. The information retention deficit for the higher-load task persisted even at 1 month post-mTBI, when behavioral performance is recovered comparable to controls. So, although the mTBI patients’ behavioral performance “normalized” at the third visit, they were still using extra effort to compensate for an information retention deficit. It is puzzling that evoked alpha power did not demonstrate a deficit in mTBIs during the first week post-injury. Based on our results, a possible explanation is that the acute injury sets off structural, metabolic, inflammatory, and oxidative processes that affect neurotransmission slowly, peaking a week after injury when they are reflected in the qEEG pattern [57]. Further investigation of this hypothesis will test the possible interpretations of acute/subacute pathophysiologies. In addition, while WM is critical for short-term memory, and short-term plasticity reflects immediate adaptation to temporary environmental changes, it is strongly linked to long-term memory formation from functional and anatomical overlap with alpha and theta oscillation involvement [58–60]. Therefore, this abnormal alpha ERD during 0-back in mTBI patients might also result in the learning impairment seen by the abnormal alpha ERD during the 2-back challenge.

Although not significant in our small study, our results that induced alpha ERD in the control group tended to be greater during 2-back compared to 0-back are consistent with previous findings that alpha ERD increases correspondingly with higher workload [61–63]. Induced alpha power has been demonstrated to increase for internal attention (inhibition of incoming sensory information that requires internal focus, motivation, or expectation, indicating greater top-down control for internal attention than for external attention [64]. However, in our study, induced alpha ERD was not greater during the 2-back task compared to the 0-back among mTBI patients, further implying that the mTBI patients were already challenged by the 0-back task and overtaxed by the 2-back. The 0-back and 2-back were presented with increasing difficulties, as in other studies. Different brain regions can be involved during different workload, which can be influenced by different pathophysiology[65]. For example, higher activation of bilateral inferior frontal gyrus pars triangularis with higher n-back workload were seen in healthy at risk for major depressive disorder individuals[66]. Further, it will be interesting to know whether or not, and how, the sequence of different workload influences the brain activity, which might be another topic to explore.

Extensive studies on alpha ERD and ERS suggest that event-related modulation of alpha power reflects sensory information gating in the cortex via selective suppression and selection [13, 67, 68]. Alpha oscillatory activities are modulated via frontothalamic loops during WM [69]. These alpha oscillations during WM actively prevent task-irrelevant stimuli from intruding on the WM buffer [70]. Alpha ERD/ERS of the frontoparietal region is known to be critical for WM [22, 71], supporting its role in top-down modulation and attention [71, 72]. Along with alpha ERD and ERS in WM, alpha oscillations of the fronto-parietal region have been demonstrated to reflect intelligence [19–21, 73]. The WM in healthy patients can be enhanced by neurofeedback training of alpha rhythms [74]. Similar neurofeedback training could potentially help mTBI patients in their recovery.

Neurometabolic changes after mTBI: the functional association between WM performance and alpha (not theta) oscillation may be related to decreased cholinergic transmission [75]. Rats subjected to mTBI show increased expression and function of the nicotinic acetylcholine receptor [76]; the acetylcholinesterase inhibitor donepezil reduces neuronal death and cognitive impairment in this model by increasing nicotinic acetylcholine-receptor activation [77]. The relationship between acetylcholine and alpha rhythms during attention/memory tasks indicate that alpha oscillations are involved in temporal coding organizations in sequential tasks similar to those we studied here [75, 78, 79], suggesting a cholinergic mechanism contributing to the learning impairment found in mTBI patients. If validated, the cholinergic mechanisms of impaired function after mTBI could be amenable to pharmacologic intervention. Even more speculative, the alpha ERD differences at the third visit, shown at Fp1 and Fp2, are localized to the orbitofrontal cortex, a location where amyloid and tau pathology is concentrated in Alzheimer’s disease (AD) [80–82], another point connecting mTBI to the known increased risk of subsequent AD and general cognitive deficits [3].

There are limitations to our study. First, the study was exploratory and, as such, was not powered for any particular comparison. Second, because of the exploratory nature of our study, multiple statistical tests were performed without adjusted significance levels; therefore, the reported p-values should be considered as support for further research into specific hypotheses rather than as conclusive evidence of associations. This is likely a common problem for complex EEG data processing due to the numerous data points that are collected. Third, it has been demonstrated that preprocessing may distort EEG signals [83]. For example, reference signal can be dynamic and inevitably affects EEG data[84]. We attempted to minimize distortion by using only widely validated pre-processing procedures [36]. Although beyond the scope of the current paper, future studies using reference electrode standardization technique should be explored[85, 86]. Finally, whereas mTBI patients are twice as likely to be male than female [1], we enrolled a similar number of males and females (7 and 6, respectively). Our experience may reflect greater altruism towards research in females and/or a greater proportion of female athletes at increased risk for mTBI [87] in our local population. Given these limitations, the fact that our findings regarding alpha power during WM task performance are consistent with previous publications is reassuring and supports further studies in larger populations over longer time courses with pre-specified hypotheses and control of type 1 error.

## Conclusions

In this pilot study, qEEG during a simple WM paradigm revealed that neurofunctionality is compromised in mTBI. The results support our hypotheses and suggest that alpha ERD and ERS differ between mTBI patients and trauma controls throughout the first month after injury. We demonstrated for the first time that frontal induced alpha ERD was marginally greater in mTBI patients during a low-work load task (0-back). Secondly, we found that induced alpha ERD for a higher-load task (2-back) did not normalize by one month after mTBI vs. trauma controls, consistent with a learning impairment reported after mTBI [34, 88]. Third, consistent with commonly reported symptoms of “foggy” or “dazed” feelings, our data show that parietal evoked alpha ERD/ERS was greater (more negative) in mTBI after two weeks. Our data notably reveals that the mTBI patients are not fully recovered at one month after injury, thus correlating EEG testing on later visits with careful residual symptom assessment may be useful. These results make it interesting to test prospectively if qEEG findings underlie the frequent post-traumatic symptoms. As the natural history and consequences of mTBI remain elusive, our results suggest that qEEG during an executive function paradigm in longitudinal studies will help identify the consequences that arise from mTBI, and have value for the diagnosis and monitoring of patients.

## Supporting information

S1 Table Induced alpha ERD during 0-back. Induced alpha ERD from all sensors during 0-back test were listed in the table, by visit and group.
(DOCX)

S2 Table Induced alpha ERS during 0-back. Induced alpha ERS from all sensors during 0-back test were listed in the table, by visit and group.
(DOCX)

S3 Table Induced alpha ERD during 2-back. Induced alpha ERD from all sensors during 2-back test were listed in the table, by visit and group.
(DOCX)

S4 Table Induced alpha ERS during 2-back. Induced alpha ERS from all sensors during 2-back test were listed in the table, by visit and group.
(DOCX)
