# Supplementary material for: Alpha desynchronization/synchronization during working memory testing is compromised in acute mild traumatic brain injury (mTBI)
Source: PLoS One. 2018 Feb 14;13(2):e0188101. doi: 10.1371/journal.pone.0188101 (PMC5812562; doi:10.1371/journal.pone.0188101)
Supplement: S1 Table — Induced alpha ERD from all sensors during 0-back test were listed in the table, by visit and group. (DOCX) [file pone.0188101.s001.docx]

| **Table S1. Induced alpha ERD during 0-back.** | | | | | | | | |  |  |  |  |  |  |  |  |  |
| --- | --- | --- | --- | --- | --- | --- | --- | --- | --- | --- | --- | --- | --- | --- | --- | --- | --- |
| Sensor | v1 | | | | |  | v2 | | | | |  | v3 | | | | |
|  | C_M_ | C_SD_ | T_M_ | T_SD_ | p-value |  | C_M_ | C_SD_ | T_M_ | T_SD_ | p-value |  | C_M_ | C_SD_ | T_M_ | T_SD_ | p-value |
| 'P3' | -1.72 | 2.01 | -2.16 | 1.83 | 0.65 |  | -1.61 | 2.12 | -1.68 | 1.86 | 0.96 |  | -1.27 | 1.11 | -1.63 | 2.00 | 0.64 |
| 'C3' | -0.75 | 1.77 | -1.73 | 1.87 | 0.28 |  | -1.15 | 1.66 | -1.19 | 1.84 | 0.96 |  | -1.21 | 0.56 | -1.49 | 2.04 | 0.65 |
| 'F3' | -0.46 | 1.42 | -1.49 | 2.18 | 0.24 |  | -0.54 | 1.65 | -0.68 | 2.57 | 0.90 |  | -1.06 | 0.52 | -1.33 | 2.73 | 0.74 |
| 'Fz' | -0.21 | 1.17 | -1.78 | 2.14 | 0.06 |  | -0.22 | 1.64 | -0.57 | 2.74 | 0.76 |  | -0.84 | 0.70 | -1.40 | 2.89 | 0.53 |
| 'F4' | -0.13 | 1.26 | -1.52 | 2.49 | 0.14 |  | -0.44 | 1.35 | -0.71 | 2.89 | 0.80 |  | -0.84 | 0.73 | -1.59 | 2.70 | 0.37 |
| 'C4' | -0.87 | 1.05 | -1.36 | 1.67 | 0.46 |  | -1.06 | 1.64 | -0.85 | 1.56 | 0.82 |  | -1.10 | 0.64 | -1.47 | 2.06 | 0.58 |
| 'P4' | -1.73 | 1.59 | -2.40 | 1.82 | 0.42 |  | -1.40 | 1.74 | -1.42 | 1.61 | 0.99 |  | -1.18 | 1.10 | -1.77 | 1.81 | 0.42 |
| 'Cz' | -0.65 | 1.37 | -1.60 | 1.68 | 0.21 |  | -0.45 | 1.66 | -0.79 | 1.85 | 0.72 |  | -0.89 | 0.57 | -1.30 | 2.43 | 0.58 |
| 'Fp1' | -1.06 | 1.72 | -2.66 | 2.16 | 0.10 |  | -1.61 | 2.03 | -0.37 | 4.48 | 0.46 |  | -1.88 | 0.52 | -2.30 | 3.28 | 0.67 |
| 'Fp2' | -0.77 | 1.56 | -2.40 | 2.29 | 0.09 |  | -1.30 | 2.08 | -0.31 | 4.23 | 0.54 |  | -1.85 | 0.40 | -2.21 | 3.08 | 0.69 |
| 'T3' | -1.31 | 1.61 | -1.65 | 1.76 | 0.68 |  | -0.95 | 1.89 | -1.50 | 1.90 | 0.60 |  | -1.35 | 0.88 | -1.68 | 1.64 | 0.59 |
| 'T5' | -1.85 | 2.02 | -2.03 | 2.23 | 0.86 |  | -1.83 | 1.71 | -1.79 | 2.13 | 0.97 |  | -2.41 | 1.51 | -2.18 | 1.75 | 0.79 |
| 'O1' | -1.82 | 2.31 | -2.72 | 2.27 | 0.43 |  | -2.42 | 1.89 | -2.33 | 2.25 | 0.94 |  | -2.39 | 1.98 | -2.49 | 2.17 | 0.92 |
| 'O2' | -1.86 | 2.23 | -2.99 | 2.06 | 0.30 |  | -2.35 | 1.97 | -2.41 | 2.31 | 0.95 |  | -2.19 | 1.17 | -2.73 | 2.19 | 0.51 |
| 'F7' | -1.18 | 1.82 | -2.74 | 2.13 | 0.12 |  | -1.40 | 1.62 | -0.64 | 3.84 | 0.59 |  | -1.64 | 0.56 | -2.28 | 3.23 | 0.51 |
| 'F8' | -0.86 | 1.45 | -2.45 | 2.38 | 0.10 |  | -1.06 | 1.50 | -0.58 | 3.31 | 0.69 |  | -1.47 | 0.59 | -2.02 | 2.95 | 0.54 |
| 'T6' | -2.26 | 1.91 | -3.13 | 1.98 | 0.37 |  | -2.75 | 2.31 | -2.41 | 2.25 | 0.79 |  | -2.92 | 1.38 | -2.49 | 2.07 | 0.62 |
| 'T4' | -1.31 | 0.97 | -1.27 | 1.35 | 0.93 |  | -0.81 | 1.69 | -0.89 | 1.60 | 0.93 |  | -1.29 | 0.65 | -1.46 | 1.82 | 0.78 |
| 'Pz' | -1.75 | 1.80 | -2.18 | 1.75 | 0.63 |  | -1.38 | 1.99 | -1.75 | 1.80 | 0.74 |  | -1.16 | 1.18 | -1.67 | 1.88 | 0.51 |

C_M_: mean for controls, T_M_: mean for mTBI, C_SD_: standard deviation for controls, T_SD_: standard deviation for mTBI. P values were calculated using two-sided t-test.
