# Supplementary material for: Alpha desynchronization/synchronization during working memory testing is compromised in acute mild traumatic brain injury (mTBI)
Source: PLoS One. 2018 Feb 14;13(2):e0188101. doi: 10.1371/journal.pone.0188101 (PMC5812562; doi:10.1371/journal.pone.0188101)
Supplement: S2 Table — Induced alpha ERS from all sensors during 0-back test were listed in the table, by visit and group. (DOCX) [file pone.0188101.s002.docx]

| **Table S2. Induced alpha ERS during 0-back.** | | | | | | | | | |  |  |  | |  |  |  |  |  |  |
| --- | --- | --- | --- | --- | --- | --- | --- | --- | --- | --- | --- | --- | --- | --- | --- | --- | --- | --- | --- |
| Sensor | v1 | | | | |  | v2 | | | | |  | v3 | | | | | |  |
|  | C_M_ | C_SD_ | T_M_ | T_SD_ | p-value |  | C_M_ | C_SD_ | T_M_ | T_SD_ | p-value |  | C_M_ | | C_SD_ | T_M_ | T_SD_ | p-value |  |
| 'P3' | 0.20 | 0.84 | -0.01 | 1.30 | 0.68 |  | 1.13 | 1.69 | 0.44 | 0.65 | 0.43 |  | 0.98 | | 0.63 | 0.35 | 1.41 | 0.21 |  |
| 'C3' | 0.65 | 0.92 | 0.34 | 1.37 | 0.58 |  | 0.82 | 1.11 | 0.54 | 0.79 | 0.63 |  | 1.05 | | 0.65 | 0.11 | 1.27 | 0.06 |  |
| 'F3' | 1.31 | 1.44 | 0.71 | 1.52 | 0.41 |  | 0.79 | 0.87 | 0.87 | 1.55 | 0.90 |  | 1.22 | | 0.95 | 0.31 | 1.43 | 0.14 |  |
| 'Fz' | 1.29 | 1.62 | 0.55 | 1.48 | 0.35 |  | 0.85 | 0.87 | 0.89 | 1.84 | 0.95 |  | 1.18 | | 0.93 | 0.43 | 1.51 | 0.23 |  |
| 'F4' | 1.02 | 1.16 | 0.33 | 1.40 | 0.28 |  | 0.66 | 0.90 | 1.13 | 1.60 | 0.47 |  | 1.27 | | 1.08 | 0.36 | 1.48 | 0.18 |  |
| 'C4' | 0.68 | 1.01 | 0.06 | 1.33 | 0.28 |  | 0.46 | 0.55 | 0.46 | 0.91 | 0.99 |  | 0.93 | | 0.98 | -0.07 | 1.21 | 0.10 |  |
| 'P4' | 0.63 | 1.12 | 0.44 | 1.42 | 0.76 |  | 1.30 | 1.98 | 0.62 | 0.63 | 0.49 |  | 1.42 | | 1.25 | 0.17 | 1.37 | 0.10 |  |
| 'Cz' | 0.63 | 0.95 | 0.46 | 1.43 | 0.76 |  | 0.92 | 0.94 | 0.46 | 1.18 | 0.42 |  | 0.97 | | 0.71 | 0.08 | 1.49 | 0.11 |  |
| 'Fp1' | 1.72 | 1.79 | 0.52 | 1.47 | 0.16 |  | 1.09 | 1.72 | 1.92 | 4.19 | 0.58 |  | 1.50 | | 1.10 | 0.40 | 1.58 | 0.12 |  |
| 'Fp2' | 1.40 | 1.30 | 0.52 | 1.57 | 0.22 |  | 1.19 | 1.68 | 1.90 | 4.09 | 0.63 |  | 1.75 | | 1.31 | 0.46 | 1.59 | 0.11 |  |
| 'T3' | 0.70 | 1.08 | 0.39 | 1.12 | 0.56 |  | 0.85 | 0.73 | 0.58 | 0.71 | 0.50 |  | 1.12 | | 0.51 | 0.16 | 1.32 | **0.04** |  |
| 'T5' | 1.46 | 1.45 | 0.52 | 0.94 | 0.16 |  | 1.45 | 1.66 | 0.88 | 0.84 | 0.50 |  | 1.37 | | 0.94 | 0.37 | 1.16 | 0.09 |  |
| 'O1' | 1.31 | 1.09 | 0.84 | 0.95 | 0.37 |  | 1.42 | 1.77 | 1.09 | 0.84 | 0.70 |  | 1.46 | | 1.25 | 0.66 | 1.49 | 0.28 |  |
| 'O2' | 1.84 | 1.96 | 1.01 | 1.36 | 0.35 |  | 1.56 | 2.21 | 0.99 | 0.74 | 0.60 |  | 2.11 | | 1.80 | 0.72 | 1.49 | 0.17 |  |
| 'F7' | 1.69 | 1.51 | 0.68 | 1.48 | 0.19 |  | 1.02 | 1.80 | 1.79 | 2.88 | 0.53 |  | 1.24 | | 0.77 | 0.34 | 1.55 | 0.12 |  |
| 'F8' | 1.50 | 1.74 | 0.38 | 1.62 | 0.20 |  | 1.04 | 1.33 | 1.37 | 2.67 | 0.75 |  | 1.45 | | 1.29 | 0.42 | 1.64 | 0.20 |  |
| 'T6' | 1.87 | 1.60 | 0.71 | 1.27 | 0.13 |  | 1.93 | 2.67 | 0.78 | 0.56 | 0.39 |  | 1.92 | | 2.01 | 0.56 | 1.43 | 0.22 |  |
| 'T4' | 0.83 | 1.14 | 0.41 | 1.20 | 0.47 |  | 0.79 | 0.98 | 0.63 | 0.70 | 0.76 |  | 0.83 | | 0.76 | -0.11 | 1.11 | 0.07 |  |
| 'Pz' | 0.50 | 0.83 | 0.19 | 1.40 | 0.56 |  | 1.09 | 1.42 | 0.59 | 0.88 | 0.49 |  | 1.16 | | 1.02 | 0.39 | 1.53 | 0.24 |  |

C_M_: mean for controls, T_M_: mean for mTBI, C_SD_: standard deviation for controls, T_SD_: standard deviation for mTBI. P values were calculated using two-sided t-test.
