# Supplementary material for: Alpha desynchronization/synchronization during working memory testing is compromised in acute mild traumatic brain injury (mTBI)
Source: PLoS One. 2018 Feb 14;13(2):e0188101. doi: 10.1371/journal.pone.0188101 (PMC5812562; doi:10.1371/journal.pone.0188101)
Supplement: S3 Table — Induced alpha ERD from all sensors during 2-back test were listed in the table, by visit and group. (DOCX) [file pone.0188101.s003.docx]

| **Table S3. Induced alpha ERD during 2-back.** | | | | | | | | | |  | |  | |  |  |  |  |  |  | |
| --- | --- | --- | --- | --- | --- | --- | --- | --- | --- | --- | --- | --- | --- | --- | --- | --- | --- | --- | --- | --- |
| Sensor | v1 | | | | |  | v2 | | | | | | |  | v3 | | | | |  |
|  | C_M_ | C_SD_ | T_M_ | T_SD_ | p-value |  | C_M_ | C_SD_ | T_M_ | | T_SD_ | | p-value |  | C_M_ | C_SD_ | T_M_ | T_SD_ | p-value |  |
| 'P3' | -2.02 | 2.72 | -2.31 | 2.50 | 0.82 |  | -1.43 | 1.58 | -1.88 | | 2.08 | | 0.64 |  | -1.68 | 1.15 | -2.16 | 2.69 | 0.60 |  |
| 'C3' | -1.95 | 2.59 | -1.73 | 1.97 | 0.85 |  | -1.32 | 1.55 | -1.80 | | 1.86 | | 0.59 |  | -1.55 | 1.11 | -2.07 | 2.24 | 0.53 |  |
| 'F3' | -1.94 | 2.49 | -1.97 | 2.56 | 0.98 |  | -0.11 | 0.73 | -1.96 | | 2.32 | | **0.03** |  | -0.87 | 1.21 | -1.96 | 2.28 | 0.21 |  |
| 'Fz' | -1.78 | 2.38 | -2.01 | 2.50 | 0.85 |  | -0.21 | 0.79 | -1.81 | | 2.19 | | **0.04** |  | -0.81 | 0.78 | -1.67 | 2.21 | 0.24 |  |
| 'F4' | -1.78 | 2.43 | -2.53 | 2.97 | 0.57 |  | -0.24 | 0.89 | -1.60 | | 2.59 | | 0.13 |  | -0.03 | 1.83 | -1.96 | 2.13 | 0.09 |  |
| 'C4' | -1.81 | 2.39 | -1.92 | 2.30 | 0.92 |  | -0.53 | 1.47 | -1.69 | | 1.87 | | 0.20 |  | -1.95 | 0.96 | -2.11 | 1.87 | 0.82 |  |
| 'P4' | -2.06 | 2.44 | -2.53 | 2.27 | 0.69 |  | -1.55 | 1.57 | -1.99 | | 2.04 | | 0.64 |  | -2.40 | 1.06 | -2.37 | 2.57 | 0.97 |  |
| 'Cz' | -1.69 | 2.69 | -1.82 | 2.33 | 0.92 |  | -0.28 | 0.58 | -1.85 | | 2.04 | | **0.03** |  | -1.83 | 1.59 | -1.98 | 2.10 | 0.88 |  |
| 'Fp1' | -1.79 | 2.56 | -2.61 | 2.96 | 0.54 |  | -0.49 | 1.11 | -2.37 | | 2.68 | | 0.06 |  | -0.46 | 1.09 | -2.31 | 2.34 | **0.04** |  |
| 'Fp2' | -1.73 | 2.60 | -2.74 | 3.12 | 0.47 |  | -0.52 | 1.13 | -2.24 | | 2.72 | | 0.09 |  | -0.37 | 1.20 | -2.32 | 2.20 | **0.03** |  |
| 'T3' | -2.00 | 2.46 | -2.17 | 2.05 | 0.88 |  | -1.09 | 1.78 | -1.52 | | 1.44 | | 0.65 |  | -1.66 | 1.58 | -2.01 | 2.29 | 0.72 |  |
| 'T5' | -2.10 | 2.54 | -2.89 | 3.02 | 0.56 |  | -1.77 | 1.98 | -2.52 | | 2.60 | | 0.54 |  | -2.32 | 1.58 | -2.86 | 3.08 | 0.63 |  |
| 'O1' | -2.22 | 2.63 | -2.88 | 2.81 | 0.62 |  | -1.16 | 2.29 | -2.54 | | 2.65 | | 0.31 |  | -2.32 | 1.59 | -3.19 | 3.05 | 0.44 |  |
| 'O2' | -2.44 | 2.63 | -3.37 | 2.43 | 0.46 |  | -1.59 | 2.59 | -3.13 | | 2.80 | | 0.31 |  | -3.03 | 1.03 | -3.48 | 2.94 | 0.64 |  |
| 'F7' | -1.84 | 2.63 | -2.67 | 2.96 | 0.55 |  | -0.35 | 1.23 | -2.75 | | 2.64 | | **0.02** |  | -0.68 | 1.28 | -2.27 | 2.69 | 0.11 |  |
| 'F8' | -1.77 | 2.40 | -2.43 | 3.10 | 0.62 |  | -0.65 | 1.05 | -2.18 | | 2.75 | | 0.12 |  | -0.52 | 1.39 | -2.14 | 2.18 | 0.09 |  |
| 'T6' | -2.92 | 2.99 | -3.71 | 2.66 | 0.58 |  | -2.50 | 2.41 | -3.21 | | 2.43 | | 0.59 |  | -3.06 | 1.42 | -3.49 | 2.58 | 0.65 |  |
| 'T4' | -2.18 | 2.34 | -2.13 | 2.22 | 0.96 |  | -2.05 | 2.53 | -1.70 | | 1.50 | | 0.78 |  | -1.30 | 0.64 | -2.01 | 1.96 | 0.26 |  |
| 'Pz' | -2.07 | 2.66 | -2.27 | 2.43 | 0.88 |  | -1.12 | 1.75 | -1.41 | | 1.57 | | 0.76 |  | -1.84 | 1.64 | -2.02 | 2.25 | 0.85 |  |

C_M_: mean for controls, T_M_: mean for mTBI, C_SD_: standard deviation for controls, T_SD_: standard deviation for mTBI. P values were calculated using two-sided t-test.
