# Supplementary material for: Alpha desynchronization/synchronization during working memory testing is compromised in acute mild traumatic brain injury (mTBI)
Source: PLoS One. 2018 Feb 14;13(2):e0188101. doi: 10.1371/journal.pone.0188101 (PMC5812562; doi:10.1371/journal.pone.0188101)
Supplement: S4 Table — Induced alpha ERS from all sensors during 2-back test were listed in the table, by visit and group. (DOCX) [file pone.0188101.s004.docx]

| **Table S4. Induced alpha ERS during 2-back.** | | | | | | | | | |  | |  | |  |  |  |  |  |  | |
| --- | --- | --- | --- | --- | --- | --- | --- | --- | --- | --- | --- | --- | --- | --- | --- | --- | --- | --- | --- | --- |
| Sensor | v1 | | | | |  | v2 | | | | | | |  | v3 | | | | |  |
|  | C_M_ | C_SD_ | T_M_ | T_SD_ | p-value |  | C_M_ | C_SD_ | T_M_ | | T_SD_ | | p-value |  | C_M_ | C_SD_ | T_M_ | T_SD_ | p-value |  |
| 'P3' | 0.57 | 1.35 | 0.07 | 1.44 | 0.47 |  | 1.05 | 1.09 | 0.71 | | 0.64 | | 0.54 |  | 0.96 | 2.07 | 0.49 | 0.77 | 0.64 |  |
| 'C3' | 0.79 | 1.95 | 0.01 | 1.38 | 0.38 |  | 1.53 | 2.12 | 0.39 | | 0.84 | | 0.30 |  | 1.80 | 3.09 | 0.41 | 0.86 | 0.38 |  |
| 'F3' | 0.97 | 2.01 | 0.19 | 1.13 | 0.38 |  | 1.08 | 0.87 | 0.75 | | 1.82 | | 0.62 |  | 1.57 | 2.36 | 0.71 | 0.99 | 0.47 |  |
| 'Fz' | 0.54 | 1.79 | 0.23 | 1.13 | 0.69 |  | 1.19 | 1.01 | 0.62 | | 1.39 | | 0.37 |  | 1.37 | 2.16 | 0.67 | 0.88 | 0.52 |  |
| 'F4' | 0.64 | 1.79 | 0.05 | 1.32 | 0.47 |  | 0.72 | 0.47 | 0.88 | | 1.63 | | 0.76 |  | 1.62 | 1.84 | 0.44 | 1.14 | 0.24 |  |
| 'C4' | -0.12 | 1.23 | -0.18 | 1.29 | 0.92 |  | 0.69 | 0.72 | 0.41 | | 0.73 | | 0.49 |  | 0.94 | 2.18 | 0.45 | 0.89 | 0.65 |  |
| 'P4' | 0.26 | 0.89 | 0.11 | 1.39 | 0.79 |  | 1.62 | 2.13 | 0.50 | | 0.62 | | 0.31 |  | 1.51 | 3.27 | 0.68 | 0.90 | 0.60 |  |
| 'Cz' | 0.31 | 1.26 | 0.00 | 1.38 | 0.63 |  | 1.04 | 0.74 | 0.51 | | 0.66 | | 0.21 |  | 1.12 | 2.52 | 0.65 | 0.92 | 0.71 |  |
| 'Fp1' | 0.95 | 1.85 | 0.49 | 1.17 | 0.57 |  | 1.79 | 1.79 | 1.26 | | 2.22 | | 0.61 |  | 1.40 | 1.78 | 0.57 | 0.89 | 0.37 |  |
| 'Fp2' | 0.90 | 1.99 | 0.52 | 1.22 | 0.66 |  | 1.79 | 1.72 | 1.32 | | 2.37 | | 0.66 |  | 1.50 | 1.64 | 0.55 | 0.87 | 0.27 |  |
| 'T3' | 0.15 | 0.66 | 0.00 | 1.40 | 0.77 |  | 0.62 | 0.43 | 0.46 | | 0.74 | | 0.60 |  | 1.22 | 2.31 | 0.22 | 0.94 | 0.40 |  |
| 'T5' | 0.65 | 1.97 | 0.53 | 1.36 | 0.89 |  | 0.88 | 0.74 | 0.41 | | 0.77 | | 0.27 |  | 1.86 | 2.57 | 0.18 | 0.98 | 0.22 |  |
| 'O1' | 0.92 | 1.56 | 1.02 | 1.47 | 0.90 |  | 2.01 | 1.39 | 1.07 | | 0.77 | | 0.21 |  | 2.10 | 3.43 | 0.73 | 1.03 | 0.43 |  |
| 'O2' | 1.18 | 1.83 | 0.55 | 1.34 | 0.45 |  | 2.20 | 2.17 | 1.02 | | 0.99 | | 0.30 |  | 2.41 | 4.08 | 0.93 | 1.08 | 0.47 |  |
| 'F7' | 1.11 | 2.09 | 0.44 | 1.22 | 0.46 |  | 1.86 | 1.88 | 0.63 | | 1.24 | | 0.23 |  | 1.26 | 1.85 | 0.60 | 0.94 | 0.49 |  |
| 'F8' | 0.91 | 1.88 | 0.46 | 1.23 | 0.59 |  | 0.90 | 0.53 | 1.02 | | 1.62 | | 0.83 |  | 1.26 | 1.93 | 0.43 | 0.83 | 0.40 |  |
| 'T6' | 0.93 | 1.34 | 0.45 | 1.25 | 0.46 |  | 1.88 | 2.27 | 0.42 | | 0.78 | | 0.23 |  | 2.08 | 3.53 | 0.52 | 1.03 | 0.38 |  |
| 'T4' | -0.09 | 1.11 | -0.21 | 1.28 | 0.83 |  | 1.00 | 1.92 | 0.29 | | 0.91 | | 0.47 |  | 0.59 | 1.51 | 0.35 | 0.92 | 0.75 |  |
| 'Pz' | 0.18 | 0.59 | 0.21 | 1.52 | 0.95 |  | 1.60 | 1.57 | 0.65 | | 0.75 | | 0.25 |  | 1.02 | 2.09 | 0.55 | 0.93 | 0.65 |  |

C_M_: mean for controls, T_M_: mean for mTBI, C_SD_: standard deviation for controls, T_SD_: standard deviation for mTBI. P values were calculated using two-sided t-test.
